# Ab interno canaloplasty for the treatment of glaucoma: a case series study

**DOI:** 10.1007/s00717-018-0416-7

**Published:** 2018-10-31

**Authors:** Norbert Körber

**Affiliations:** Augencentrum Köln-Porz, Josefstraße 14, 51143 Cologne, Germany

**Keywords:** Canaloplasty, ABiC, Combined operation, POAG, Kanaloplastik, ABiC, Kombinierte Operation, POAG

## Abstract

**Purpose:**

To describe and evaluate the efficacy of ab interno canaloplasty (ABiC) in patients with primary open-angle glaucoma (POAG).

**Methods:**

This single-center consecutive case series study included patients with cataract and open-angle glaucoma (combined procedure) and pseudophakic patients (mean age, 76 years; range, 66–83 years) with POAG who underwent ABiC using the iTrack™ 250-μm microcatheter (Ellex Medical Lasers Pty Ltd, Adelaide, Australia) to circumferentially viscodilate and intubate Schlemm’s canal without placement of a tensioning suture. The primary endpoints were mean intraocular pressure (IOP) and mean number of glaucoma medications at 1, 3, 6, 9, and 12 months postoperatively.

**Results:**

In total, 20 patients (20 eyes) were enrolled in the study. Mean IOP reduced from 18.5 ± 3.44 mm Hg preoperatively to 14.88 ± 2.82 mm Hg (*n* = 17), 13.80 ± 2.05 (*n* = 12), 14.57 ± 2.59 mm Hg (*n* = 9) and 15.47 ± 2.42 (*n* = 6) at 1, 3, 6 and 9 months postoperatively. The 12‑month data for two patients showed that IOP had reduced from 17 mm Hg preoperatively to 16 mm Hg in one patient and from 20 mm Hg to 13 mm Hg in the other patient. The mean number of medications was reduced from 2.4 preoperatively to 0.25 at the last follow-up visit. There was one reported complication of limited descemetolysis near the limbus by the viscoelastic during the dilatation of Schlemm’s canal. No adverse events as a result of the device were reported.

**Conclusions:**

ABiC was straightforward to perform in this group of patients with minimal complications. Although initial findings from this study indicate that ABiC is comparable to conventional canaloplasty in lowering IOP and medication dependency, long-term follow-up in a large patient cohort is required to confirm the efficacy of this minimally invasive glaucoma procedure.

## Zusammenfassung

**Zweck:**

Die Wirksamkeit und die Durchführung der Kanaloplastik ab interno (ABiC) bei Patienten mit primärem Offenwinkelglaukom werden beschrieben.

**Methodik:**

Diese konsekutive Einzelzentrum-Fallstudie schließt Patienten mit Katarakt und Offenwinkelglaukom (kombinierte Operation) und Patienten mit Pseudophakie und Offenwinkelglaukom (POAG) ein, bei denen eine ABiC mit dem iTrack™ 250-μm-Mikrokatheter (Fa. Ellex Medical Lasers Pty Ltd, Adelaide, Australien) durchgeführt wurde, um so den Schlemm-Kanal zirkulär zu viskodilatieren und zu sondieren, ohne eine Spannnaht zu implantieren (mittleres Alter: 76 Jahre, Spannbreite: 66–83 Jahre). Die primären Endpunkte waren der Intraokulardruck (IOP) und die mittlere Zahl von Glaukommedikamenten 1, 3, 6, 9 und 12 Monate postoperativ.

**Ergebnisse:**

Zwanzig Patienten (20 Augen) wurden in die Studie eingeschlossen. Der mittlere IOP wurde von 18,5 ± 3,44 präoperativ auf 14,88 ± 2,82 mmHg (*n* = 17), 13,80 ± 2,05 (*n* = 12), 14,57 ± 2,59 mmHg und 15,47 ± 2,42 (*n* = 6) nach 1, 3, 6 und 9 Monaten postoperativ reduziert. Die 12-Monats-Daten für 2 Patienten ergaben eine Reduktion des IOP von 17 mmHg präoperativ auf 16 mmHg bei dem einen Patienten und von 20 mmHg auf 13 mmHg bei dem anderen Patienten. Die mittlere Zahl an Glaukommedikamenten wurde von 2,4 präoperativ auf 0,25 bei der letzten Vorstellung im Rahmen der Nachbeobachtung reduziert. Es wurde eine Komplikation verzeichnet, die aus einer begrenzten Descemetolyse limbusnah bestand, die durch Austritt von Viskoelastikum aus dem Schlemm-Kanal während der Dilatation entstand. Komplikationen durch die Anwendung des iTrack wurden nicht beschrieben.

**Schlussfolgerungen:**

ABiC konnte bei dieser Gruppe von Patienten ohne Probleme mit minimalen Komplikationen durchgeführt werden. Obwohl die initialen Ergebnisse dieser Studie zeigen, dass ABiC den IOP und die Medikation vergleichbar mit der konventionellen Kanaloplastik senkt, bedarf es einer länger dauernden Untersuchung bei einer größeren Patientengruppe, um die Effizienz dieser minimalinvasiven Glaukomoperation zu belegen.

## Introduction

Primary open-angle glaucoma (POAG) is a sight-threatening disorder caused by inadequate ocular outflow, leading to elevated intraocular pressure (IOP).

There are a number of surgical treatment options for POAG. Traditionally, these have included aqueous shunts and trabeculectomy, which, although effective, are associated with numerous side effects including bleb leaks, cataracts, blebitis, endophthalmitis, and vision loss [1–3].

Perhaps not surprisingly, glaucoma surgeons are increasingly turning to non-penetrating and/or bleb-independent procedures such as the Trabectome®, the iStent®, and an investigational device known as the Hydrus Microstent™. However, while safer than conventional glaucoma surgery, these minimally invasive glaucoma surgery (MIGS) options are usually performed alongside cataract surgery to confer maximum benefit but appear to be less effective, with patients often requiring medical therapy to maintain their IOP [4–6]. This is likely explained by the fact that each of these approaches fails to address all aspects of the ocular outflow system [7, 8].^.^By contrast, canaloplasty, a minimally invasive glaucoma treatment that addresses all aspects of the ocular outflow system, i. e., the trabecular meshwork, Schlemm’s canal, and the distal collector channels, is not only safe, but has also been shown to be as effective as filtering surgery both in terms of IOP-lowering effects and reducing dependence on glaucoma medication [9, 10]. Canaloplasty is also effective with or without cataract surgery [11–13].

An evolution of viscocanalostomy, traditional canaloplasty employs circumferential (360°) catheterization of Schlemm’s canal along with gentle viscodilation. This breaks adhesions within the canal and allows the compressed tissue planes of the trabecular meshwork and sclera to separate and any herniated trabecular meshwork tissue to withdraw from collector channels. Traditional canaloplasty employs placement of a 9-0 or 10-0 Prolene tensioning suture/stent to ensure the patency of Schlemm’s canal. However, a review of 3‑year data by Lewis and colleagues indicated that 360° viscodilation alone, i. e., canaloplasty without a suture, successfully lowered IOP [11]. A study by Susan Senft, MD, also showed that the presence or absence of a suture did not affect outcomes such as visual acuity, IOP, visual field parameters, and number of medications required to maintain IOP [14].

Approved by the United States Food and Drug Administration, suture-free canaloplasty, known as ab interno canaloplasty (ABiC), is gaining popularity with glaucoma surgeons across Europe and the United States as a treatment for mild-to-moderate POAG, mainly thanks to its ease of use, comprehensive approach, and low risk profile. ABiC also spares conjunctival manipulation, which means that future procedures can be performed if necessary [15] and is the only MIGS to address all aspects of ocular outflow.

Since ABiC is a relatively new development of traditional canaloplasty, as yet there are no published studies describing the procedure and few data describing clinical outcomes. The aim of this case series study, therefore, was twofold: to describe and evaluate the surgical procedure, and to assess the ability of ABiC to lower IOP and reduce dependence on medication in patients with POAG.

## Methods

### Study design and patients

This was a single-center consecutive case series study designed to evaluate the efficacy of ABiC in reducing IOP and dependence on glaucoma medication at 1, 3, 6, 9, and 12 months postoperatively. Secondary endpoints included surgical and postsurgical complications. All surgeries were undertaken at Augencentrum Köln-Porz and performed by a single surgeon (NK).

The study was performed in accordance with the principles stated in the Declaration of Helsinki and its amendments, and all patients provided written informed consent.

Inclusion criteria were a minimum age of 18 years, cataract or pseudophakia, and a diagnosis of controlled POAG or exfoliative glaucoma. Phakic patients and those with neovascular disease, uveitis, peripheral anterior synechiae, as well as angle-closure, narrow-angle, neovascular, posttraumatic, and other forms of secondary glaucomas were not eligible for inclusion in the study.

### Clinical examinations

Ophthalmic examinations including medication use, IOP, slit-lamp, and fundus examination were performed at baseline and at 1 day, 1, 3, 6, 9, and 12 months postoperatively. Gonioscopy was performed at baseline, 1, 3, 6, 9, and 12 months. A complete medical history was also taken before patients underwent the procedure.

### Surgical procedure

**Fig. 1:**
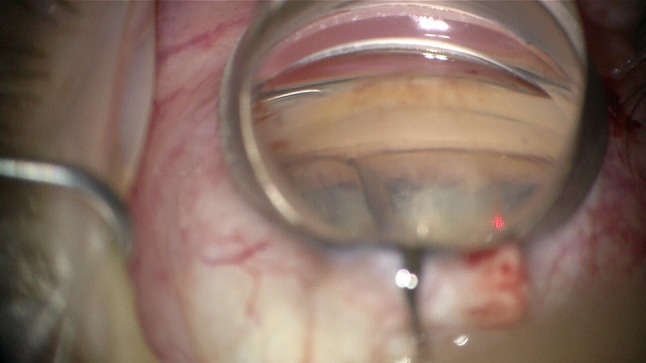
Catheter tip introduced into Schlemm’s canal. Tip location visible by red light shining through the trabecular meshwork

**Fig. 2:**
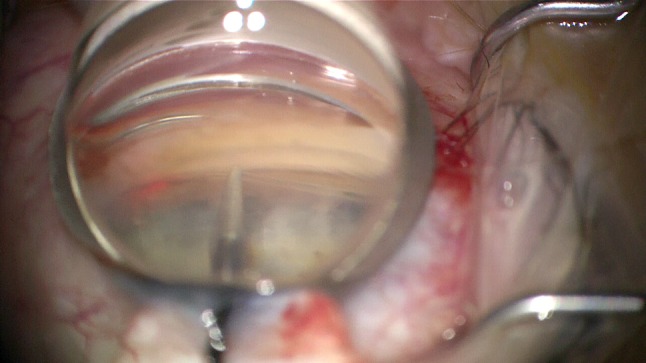
A 24-G needle in location for the incision of the trabecular meshwork. Incision location: transit from pigmented to nonpigmented meshwork

All patients underwent ABiC under local anesthesia consisting of retrobulbar injection of Carbocaine and Lidocaine. The iTrack™ 250-μm microcatheter (Ellex Medical Lasers Pty Ltd., Adelaide, Australia) with a fiber-optic light and lumen was passed through a clear corneal incision (1.8 mm in pseudophakic eyes and 2.4 mm in combined cases) and then through a small opening in the trabecular meshwork in order to circumferentially viscodilate and intubate Schlemm’s canal (Fig. 1). The meshwork opening was made by utilizing a 24-G needle with a 20° bent tip (Fig. 2). Precisely controlled delivery of Healon/Healon GV during advancement and withdrawal of the catheter allowed the compressed tissue planes of the trabecular meshwork to separate, and any herniated inner wall tissue to withdraw from the collector channels. In combined cases, phacoemulsification and IOL implantation were performed prior to ABiC. At the end of the procedure, Healon was removed from the anterior chamber and the pupil was constricted using a small dose of Miochol. A subconjunctival dose of gentamycin and dexamethasone was then applied.

Postoperatively, patients received combined drops of gentamycin and dexamethasone four times daily for 1 week followed by diclofenac drops four times daily for 1 month.

## Results

In total, 20 eyes (11 OS, 9 OD) of 20 patients (mean age, 76 ± 5.79 years; 10 male: 7 female) were enrolled in the study.

Preoperatively, mean IOP (all eyes) was 18.5 ± 3.44 mm Hg. Mean IOP at 1, 3, 6, and 9 months was 14.88 ± 2.82 mm Hg (*n* = 17), 13.80 ± 2.05 (*n* = 12), 14.57 ± 2.59 mm Hg (*n* = 9), and 15.47 ± 2.42 (*n* = 6), respectively (Table 1). These data correspond to reductions in IOP of 19.57%, 25.41%, 21.25%, and 16.38% at 1, 3, 6, and 9 months postoperatively. Additionally, 12-month data for two patients showed that IOP had reduced from 17 mm Hg preoperatively to 16 mm Hg in one patient and from 20 to 13 mm Hg in the other patient.

**Table 1:** Reductions in mean IOP

Examination	*n*	Mean IOP (mm Hg) ± SD
Preoperative	20	18.5 ± 3.44
1 Month	17	14.88 ± 2.82
3 Months	12	13.80 ± 2.05
6 Months	9	14.57 ± 2.59
9 Months	6	15.47 ± 2.42
12 Months	2	14 ± 2.12

*IOP* intraocular pressure, *SD* standard deviation

A reduction in dependence on antiglaucoma medications was also noted. Preoperatively, all patients required at least one medication, with four of 20 (20%) patients requiring three medications to control their IOP. At the last follow-up visit, only four of 20 (20%) patients still required antiglaucoma medication (one medication each) versus 100% of patients preoperatively (Table 2). Overall, considering the number of drops administered combined with the number of different medications used, mean medication use reduced from 2.4 preoperatively to 0.25 at the last follow-up visit.

**Table 2:** Reduction in medication use

Eye	Preoperative medication (*n*)	Postoperative medication (*n*)
1	1	1
2	1	0
3	3	1
4	2	0
5	3	0
6	1	0
7	1	0
8	2	0
9	1	0
10	3	0
11	2	0
12	3	0
13	1	0
14	1	1
15	1	0
16	1	0
17	1	1
18	1	0
19	1	0
20	2	0

*n* number of medications

There was one complication, i. e., limited descemetolysis near the limbus by the viscoelastic during the dilatation of Schlemm’s canal. No adverse events as a result of the iTrack™ device were reported.

## Discussion

Although trabeculectomy and aqueous tube shunts are still the mainstays of glaucoma treatment with their efficiency in terms of IOP reduction compared with canaloplasty [16–19], there appears to be a growing interest in bleb-free, minimally invasive procedures that offer a better safety profile. However, MIGS such as the Trabectome® and the iStent® are not always effective, particularly when performed as standalone procedures [4–6]. By contrast, a number of peer-reviewed studies show that canaloplasty is not only minimally invasive, but also maximally effective. ABiC works in the same way as traditional canaloplasty, but does not employ a tensioning suture; thus, it may be considered as angioplasty without a stent. ABiC also differs from traditional canaloplasty in that the iTRACK™ microcatheter is inserted through either a clear corneal or a limbal micro-incision, and requires the creation of a scleral flap.

Since ABiC is a relatively new development of traditional Canaloplasty, there are few published data describing its IOP-lowering effects or its ability to reduce dependence on medications. In this case series study, there were reductions in IOP of between 16.38 and 25.41% through 9 months postoperatively. Additionally, we found that dependence on medication was significantly reduced; at the last follow-up visit, only 20% of patients still required medication, versus 100% before the procedure.

It somewhat challenging to compare these findings with those of other studies, since there are few published data describing outcomes following ABiC. However, results from Lewis and coworkers’ landmark study of Canaloplasty showed that mean postoperative IOP for all eyes (*n* = 157) decreased from 23.8 ± 5.0 mm Hg on 1.8 ± 0.9 medications preoperatively to 15.2 ± 3.5 mm Hg on 0.8 ± 0.9 medications at 3 years postoperatively. Of these patients, approximately 15% did not receive a tensioning suture [11]. Additionally, data from an ongoing case series study by Mark J. Gallardo, MD, and Mahmoud A. Khaimi, MD, who evaluated ABiC with and without phacoemulsification showed that for all eyes (*n* = 228) there was a marked reduction in mean IOP (27.9%) and number of medications (50%) at 6 months postoperatively (*n* = 52). A subanalysis showed that in eyes receiving ABiC combined with cataract surgery (*n* = 127), there was a 23.52% reduction in mean IOP and a 100% reduction in number of medications at the 6‑month visit (*n* = 34). When ABiC was performed as a standalone procedure in pseudophakic patients, data from Dr. Khaimi’s and Dr. Gallardo’s studies combined (*n* = 83) showed there was a total average decrease of 33.48% in mean IOP and 66.66% in mean medication use at 6 months postoperatively (*n* = 18; [20]). Our own findings are on a par with these data, suggesting that ABiC produces consistent outcomes and does not need to be performed alongside cataract surgery in order to be effective. The present findings are also in line with outcomes following traditional canaloplasty, which show that IOP is consistently lowered to the mid-teens [9, 21, 22].

The second aim of the present study was to evaluate the surgical procedure and to assess the nature and incidence of complications. In the present study, there was only one complication—limited descemetolysis near the limbus by the viscoelastic during the dilatation of Schlemm’s canal. Although there were no cases of hyphema in the present study, surgeons should be aware that this may occur following canaloplasty/ABiC. While some surgeons may be concerned about this, it is actually a positive sign, confirming that there is connection between the anterior chamber and the aqueous into the outflow system [23, 24]. Surgeons may also find that, on occasion, they encounter a collapsed Schlemm’s canal. Occasionally (about 5% of the cases), the catheter trails off into a collector channel. However, this is easily overcome. Because the iTRACK-250™ microcatheter is so long, the surgeon is able to make a paracentesis 180° away and start to catheterize in the opposite direction. Doing so should lead to successful 360° viscodilation. There may also be rare occasions where the catheter is misdirected, for example, it may go into the suprachoroidal space. However, the tip is blunt so there is no risk of damage to the choroid. The illuminated tip also advises of the catheter’s location. Catheter misdirection may be resolved by applying pressure to the affected area or, again, by making a paracentesis 180° away from the site of the misdirection and catheterizing in the opposite direction.

Surgeons new to canaloplasty/ABiC may be somewhat nervous about the technique, particularly those who have never performed viscocanalostomy. One of the main challenges with traditional canaloplasty was placing a tensioning suture, an issue negated by ABiC. There is no doubt that both traditional canaloplasty and ABiC have a learning curve, but, typically, a surgical fellow is comfortable with ABiC after only five to ten procedures. An experienced glaucoma surgeon may achieve familiarity with the procedure much sooner. Moreover, making the transition to ABiC is easier now than it was when it was first introduced 10 years ago; since that time, we have become more familiar with the procedure and a certain consensus has emerged about the best way to achieve consistently excellent results with canaloplasty or ABiC.

Clearly, the present study has limitations including the small study cohort. Additionally, follow-up data beyond 6 months postoperative were only available for a handful of patients.

However, findings from this small study indicate that ABiC lowers IOP and medication dependency comparable to conventional canaloplasty and other studies describing outcomes following ABiC. The procedure was also straightforward to perform in this group of patients, with minimal complications. Nevertheless, long-term follow-up in a large patient cohort is required to confirm the efficacy of this minimally invasive glaucoma procedure.
